# Book Review: Pedagogies and Policies for Publishing Research in English: Local Initiatives Supporting International Scholars

**DOI:** 10.3389/fpsyg.2021.697603

**Published:** 2021-06-09

**Authors:** Xiaolong Cheng, Lawrence Jun Zhang

**Affiliations:** ^1^School of Foreign Languages, Hubei University of Technology, Wuhan, China; ^2^Faculty of Education and Social Work, University of Auckland, Auckland, New Zealand

**Keywords:** academic publishing, English as an additional language, pluralingual/multilingual writers, international publishing, English for publication purposes

James Corcoran, Karen Englander, and Laura-Mihaela Muresan (New York, NY: Routledge), 2019, 299 pages, ISBN 9781138558090

With the globalization and internationalization, English has become a lingua franca in the modern world, and it serves a significant role in publicizing research findings for international communities (Schreier et al., 2020; Cheng and Zhang, 2021). To establish the authority identity in academia around the world, there is a pressing need for scholars outside Anglophone center contexts to disseminate their research in English. Such a need places great demands on English-as-an-additional-language (EAL) researchers, as they are confronted with a variety of obstacles in their attempt to publish studies in English (Mu and Zhang, 2018). Against this background, Corcoran, Englander, and Muresan's book, *Pedagogies and Policies for Publishing Research in English*, is a welcome and timely addition to the extant literature in this filed. By examining the pedagogical initiatives in diverse contexts, this volume is poised to provide scholars with practical guidance and recommendations, which would smooth their journey of writing in English for international publication.

This book starts with an introductory chapter, co-authored by the three editors. In this chapter, they establish the rationale for their work, explicate the aims of this volume, offer reasons for employing a pluralistic approach to such a volume, and detail the difficulties facing pluralistic/multilingual writers while they publish in English. In what follows, we review and evaluate the 17 chapters that are arranged according to the geolinguistic contexts: Latin America, Northern Europe, Eastern and Southern Europe, East Asia, South Asia, Africa, and Persian Gulf.

Part 1 (Chapters 2–4) focuses on the local support for researchers in Latin America. Chapter 2 written by Janssen and Restrepo introduces a tutoring service for publishing in English in a Colombian university, which is designed to enhance the academic and research production. In this service, novice EAL writers are offered hand-on suggestions and guidelines from experienced researchers, which can help them to understand ways of disseminating their research internationally. In Chapter 3, Encinas-Prudencío, Sánchez-Hernández, Thomas-Ruzic, Cuatlapantzi-Pichón, and Aguilar-González reports a longitudinal case study that investigated the factors which constrained, or helped promote, EAL teachers' scholarly English publication. They report that collaboration, mentoring network, and workplace demands have a great influence on such teachers' research publishing practices in their trajectories toward authorship. Chapter 4 (Waigandt, Noceti, and Lothringer) reviews and comments on four institutional policies implemented to satisfy research students' and scholars' needs of having their research published in English.

Part 2 looks at the initiatives supporting scholars in Northern Europe. In Chapter 5, Arnbjörnsdóttir reveals, through a mixed-methods study, the challenges confronted by Icelandic scholars (e.g., inadequate English writing ability and the lack of genre awareness). Accordingly, the contributor introduces a research-driven English for research publication purposes (ERPP) workshop. As regards the supporting policies available to Norwegian researchers, Muir and Solli in Chapter 6 investigate the relevant initiatives and put emphasis on new instructional approaches to emerging scholars' academic writing.

Part 3 addresses the scholarly writing for researchers based in Eastern and Southern Europe. Chapter 7 opens this part, where Muresan and Pérez-Llantada offer a case study on a veteran researcher's development in academic literacy and competence. Furthermore, the authors highlight that in order to get published globally, plurilingual writers should read and write extensively, and be aware of the rhetoric and linguistic characteristics of English texts. In Chapter 8, Burgess, Martín, and Balasanyan elaborate on an ERPP course for doctoral students, which takes a critical pragmatic approach to teach research writing. Such a course affords a better understanding of scholarly publication in English.

Parts 4 and 5 turn to the support for researchers in East Asia and South Asia, respectively. Chapter 9 by Li and Cargill characterizes and details a “master-class” methodology, which empowers English for academic purposes (EAP) teachers to impart their students the skills and knowledge in relation to English publishing in China. Administering a large-scale questionnaire, Zheng and Cao in Chapter 10 explore EAL researchers' ERRP beliefs and practices. They find that the majority of respondents believe it is equally important for researchers to publish in English and Chinese. As a sole contribution, Chapter 11 by Nauman represents the region of South Asia. In this chapter, the contributor discusses the effects of English language teaching reforms carried out by the Higher Education Commission on Pakistani researchers' language and research skills. He ends this chapter with suggestions on how to meet such scholars' publishing needs in their professional career.

Part 6 is specific to the pedagogies and initiatives for publication in Africa. Chapter 12 drafted by Messekher and Miliani identifies the difficulties and obstacles that novice scholars encounter while they seek to publish their theses in the form of journal articles. To ease their publishing journey, the contributors recommend a grounded approach to academic research writing, which aims to prepare Algerian emerging scholars for publishing their studies in international journals. In Chapter 13, Omobowale, Akanle, and Akinsete look into the colonizing effects of English on research publication in Nigeria. The three authors point out that Nigerian English is in an unrecognized status and considered non-standard. As such, Nigerian scholars are disadvantaged while intending to publicize their research in English journals with high quality and earn recognition internationally.

The final part (Part 7) shifts attention to the publishing practices in the Persian Gulf. Shahriari and Ghonsooly in Chapter 14 begin this part, delving into the status quo of research publication in Iran. They show a range of challenges that novice and experienced scholars encounter, including the potential biases against submissions from Iranian researchers, the limited access to the research resources, and the lack of a solid academic network. Chapter 15 (Mirhosseini and Shafiee) identifies a tendency that Iranian researchers have to publish their studies in English journals with high impact factors due to the policies valuing and encouraging English publication. Consequently, the researchers in Iran bear great pressure with regard to producing English research articles. The following chapter (Chapter 16 by Nunn and Deveci) presents an instructional approach to scholarly writing: “holistic argumentation creation.” With such an approach, research students have opportunities to foster their awareness of academic writing conventions and enhance their ability of research writing. This part is concluded by Swales with a short envoi. In this envoi, he argues that native English-speaking researchers are also faced with constraints and challenges in the course of publishing in English. In addition, he provides EAL scholars with recommendations, which are expected to benefit their English publishing practices.

This whole collection, which is different from the previous books on the same topic that focus on research writing itself (e.g., Oliver, 2014; Parija and Kate, 2018), brings the policies and initiatives for plurilingual researchers' writing for publication to the fore. In reading this collection, we have found several commendable features. First, this volume is characterized by its coverage of possible themes. Specifically, it encompasses 17 chapters, spanning different themes such as the polices supporting English writing for publication in different local communities (e.g., Chapter 4 and 6), the initiatives for conserving local languages and developing EAL scholars' bi- or multi-literacies (e.g., Chapter 7 and 10), the challenge of the privileging use of English to convey scientific knowledge (e.g., Chapter 13 and 15), and the social practices enabling emerging scholars to participant in academic activities and enhance their research writing (e.g., Chapter 2 and 5).

Another strength that merits mention is the breadth of contexts. This volume introduces and describes the pedagogical writing interventions and policies for English publishing in seven different geolinguistic regions outside the Anglophone center. As the introductory chapter points out, much attention has been paid to how researchers publish their work in native English-speaking contexts because of the hegemony of English in transmitting knowledge for global academia. Thus, such a volume not only fills an important niche, but also provides us with a global perspective on and deep insight into how EAL researchers are scaffolded to publish research articles in English outside English-as-a-native-language areas. We can compare and understand the similarities and differences of these policies, which are implemented in different EAL regions. This would offer important implications for research practitioners and policy-makers. Also, it should be noted that the editors of this volume include areas such as South Asia and Africa. Due to the economic and politic disadvantages, such regions are often underrepresented and under-researched in the current literature. As a result, this volume makes a contribution with regard to advancing our existing knowledge about the endeavors made by these regions to improve non-native English-speaking writers' publication.

Furthermore, what we should emphasize is that the contributors are mainly novice and less prolific scholars, rather than experienced and renowned researchers. In general, such scholars are in a relatively disadvantaged position vis-à-vis their established counterparts. Consequently, their voices about publishing research articles remain unheard. Considering that they represent the majority of front-line researchers, their stories, experiences, and initiatives of getting their research published in English academic journals should be heard. In this sense, we should commend the editors in that they have successfully provided a platform for such researchers to raise their voices in the mainstream scholarship and make their efforts in trajectories toward authorship visible to the international academic community.

Finally, this volume should be praised because of its unique arrangement or design of its contents. Prior to the elaboration on each chapter in the seven EAL regions, the editors have provided a guide to readers. Such a brief introduction prompts readers to understand the main points of each chapter easily, making this collection extremely accessible to readers. Various tables, figures, and examples deserve mention as well, since they enable readers to catch the gist in each chapter promptly.

The volume would have been more comprehensive had been added contributions from Japan and South Korea in East Asia. Although they are EAL countries, their researchers actively participate in academic conferences and express their thoughts in influential English-language research journals. Therefore, it would have been a perfect collection if their pedagogies and polices for scaffolding the researchers' English publishing practices were included, which may generate useful information for other similar EAL countries and contexts. Besides, more pages could have been devoted to the comparison of the difficulties between native and non-native English-speaking researchers while writing for English publication. Given that English publishing is a demanding undertaking and imposes great pressure on both native and non-native English-speaking writers, these two groups of researchers possibly share similar challenges and the native/non-native dichotomy probably is not the only reason for their success/failure in the enterprise of scholarly publication.

These minor limitations do not dim the contributions made by such a volume and it is worth a wide readership, including novice/experienced researchers, course designers, pedagogues, higher institution administrators, and policy-makers. To facilitate the use of this book, we would like to attempt to offer some advice for emerging researchers. Firstly, they should foster a habit of self-reflection on their publishing practices, especially the difficulties and challenges that they face. In doing so, they can garner suitable and targeted solutions from this book, which can make them benefit from this volume and improve the effectiveness. Additionally, since such a book provides them with feasible and general suggestions from experienced researchers, including how to convert their PhD theses into journal articles and how to become veteran researchers, among other things, it is imperative that emerging scholars incorporate them into their normal English scholarly publishing activities. Doing so probably will enable them to have a better understanding of such valuable recommendations. Lastly, they need to make clear about the features of the contexts for their publishing practices, as some guidelines and principles offered in this book are context-specific. In this sense, they should not follow them uncritically. Instead, they need to adjust such guidance according to their own distinctive contextual needs in order to maximize the value of this book.

## Author Contributions

XC wrote the first draft. LZ revised the first draft. Both authors approved the revisions.

## Conflict of Interest

The authors declare that the research was conducted in the absence of any commercial or financial relationships that could be construed as a potential conflict of interest.
